# Investigation of cholecystokinin-8s-induced hypophagia in neonatal broiler chicks: Roles of central adrenergic and serotonergic systems

**DOI:** 10.1016/j.ibneur.2025.12.011

**Published:** 2025-12-22

**Authors:** Hamed Zarei, Ghazaleh Mahdavi, Emad Shahrabi, Keyvan Hasani

**Affiliations:** a Department of Biology, CT.C., Islamic Azad University, Tehran, Iran; b Department of Basic Sciences of Veterinary Medicine, Garmsar Branch, Islamic Azad University, Garmsar, Iran

**Keywords:** Adrenergic, Broiler, Cholecystokinin, Food intake, Intracerebroventricular injection, Serotonergic

## Abstract

Cholecystokinin (CCK) is integral to the central control of appetite, with its sulfated octapeptide isoform (CCK-8s) being particularly relevant. While CCK's anorexigenic effects have been established, the interactions between CCK-8s and adrenergic and serotonergic receptor subtypes in mediating food intake remain incompletely understood. This research was designed to examine the impact of centrally administered CCK-8s on food consumption in broilers, focusing on how it interacts with adrenergic and serotonergic receptors to influence intake. For this purpose, nine experiments were conducted on broilers. In experiment 1, broilers received central infusion of saline and CCK-8s (0.25, 0.5, and 1 nmol). The second experiment evaluated the effects of saline, the α1-adrenergic antagonist prazosin, CCK-8s at 1 nmol, and a combination of prazosin with CCK-8s. Experiments three through nine followed a similar design, substituting prazosin with other agents targeting different receptors, including the α2 antagonist yohimbine, β1 antagonist metoprolol, β2 antagonist ICI 118,551, serotonin synthesis inhibitor PCPA, serotonin reuptake inhibitor fluoxetine, 5-HT1A receptor agonist 8-OH-DPAT, and 5-HT2C receptor antagonist SB242084. Food intake was recorded cumulatively up to two hours after infusions. The outcomes demonstrated that CCK-8s (0.5 and 1 nmol) significantly decreased meal intake compared to controls (P < 0.05). Interestingly, administration of CCK-8s with fluoxetine significantly enhanced its appetite-suppressing effect (P < 0.05), whereas co-injections with SB242084, PCPA, or ICI 118,551 decreased this effect (P < 0.05). The data suggest that the anorexigenic influence of CCK-8s involves signaling pathways mediated by β2 adrenergic and 5-HT2C serotonergic receptors, offering fresh perspectives on neuropeptidergic regulation of feeding and highlighting the need for further detailed mechanistic studies.

## Highlights

• Central CCK-8s (0.5 & 1 nmol) induces significant hypophagia in broiler chicks.
• β2-adrenergic receptor antagonist ICI 118,551 attenuates CCK-8s hypophagia.
• 5-HT2C receptor antagonist SB242084 reduces CCK-8s anorexigenic effects.
• Serotonin reuptake inhibitor fluoxetine enhances CCK-8s-induced hypophagia.

## Graphical Abstract

**Figure:**
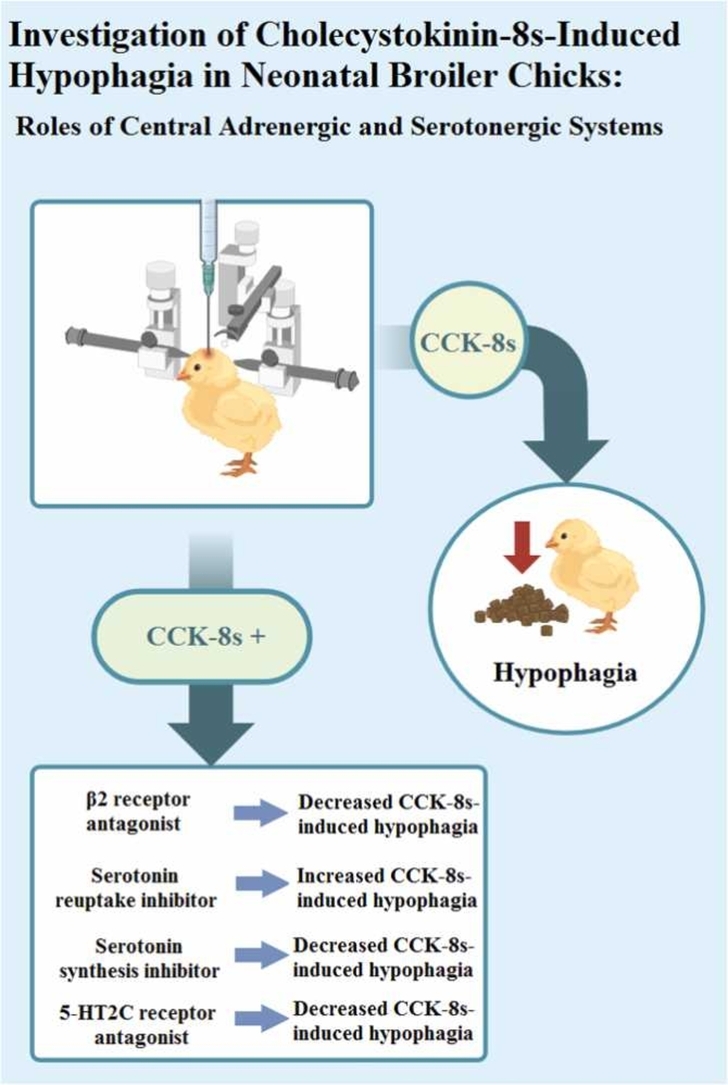

## 1 Introduction

Feed intake regulation is a fundamental physiological process conserved across vertebrates, including birds and mammals. This process relies on intricate neural and endocrine networks that integrate peripheral and central signals to maintain energy balance (Cifuentes and Acosta, 2022). In avian species, notably broiler chickens selected for rapid growth, food intake control is critical not only for optimizing production efficiency but also for ensuring animal health and welfare (Jie et al., 2024). The hypothalamus has been identified as the central hub for appetite regulation in birds, analogous to mammals, integrating hormonal, neurotransmitter, and nutrient signals to modulate feeding behavior (Rahmani et al., 2021, Mahdavi et al., 2023). Understanding food consumption regulation in broilers is particularly important due to breed-specific alterations in central appetite pathways induced by genetic selection for meat yield, which may influence responsiveness to satiety signals (Mahdavi et al., 2024). Investigating neurochemical mediators and their central pathways opens avenues for advancing knowledge on appetite control mechanisms in poultry, with implications for improving growth management and reducing metabolic disorders in broiler production.

Cholecystokinin (CCK), traditionally recognized as a key gastrointestinal hormone, is also among the most prevalent neuropeptides within the central nervous system (CNS) of mammals and avian species alike, including chickens (Tachibana and Tsutsui, 2016). Among its various biologically active isoforms, including CCK-33, the sulfated octapeptide CCK (CCK(26−33), CCK-8s), and the tetrapeptide CCK (CCK(30−33), CCK-4), CCK-8s is the most extensively studied due to its potent physiological effects (Rehfeld, 2021). CCK interacts with distinct receptor subtypes, which are classified into two main categories: CCKA (also known as CCK1) and CCKB (also known as CCK2) (Staljanssens et al., 2011). Peripherally, CCK modulates several digestive processes, including gallbladder contraction, gastrointestinal motility, pancreatic enzyme release, regulation of gastric emptying, and acid secretion (Rehfeld, 2021, Williams, 2019). Beyond its peripheral roles, CCK-8s functions centrally as an anorexigenic neurotransmitter, influencing feeding behavior. In rodent models, peripheral infusion of CCK suppresses meal intake (Abdalla, 2017, Asin et al., 1992), and similarly, in chicks, intraperitoneal (IP) infusion of CCK-8s remarkably reduce feeding, whereas CCK-4 does not elicit such effects (Tachibana et al., 2012). Notably, central administration of CCK-8s via intracerebroventricular (ICV) injection induces pronounced hypophagia in chicks (Ebrahimnejad et al., 2025). This evidence underscores the importance of elucidating the central pathways mediating CCK-8s-induced anorexia in broilers.

The adrenergic system represents a crucial neural mechanism that substantially contributes to controlling food intake and sustaining energy balance. Norepinephrine (NE) is a key catecholamine neurotransmitter within the CNS. NE mediates its physiological effects predominantly via activation of adrenergic receptors, which are classified into α1, α2, β1, β2, and β3 subtypes (Wu et al., 2021). The adrenergic neural pathways within the avian brain have been implicated in the control of meal consumption, as evidenced by studies showing that ICV administration of NE into the hypothalamic paraventricular nucleus (PVN) stimulates meal consumption in chickens (Yousefvand and Hamidi, 2020). This orexigenic response is primarily mediated via α₂-adrenergic receptors, since activation of these receptors by agonists such as clonidine increases feeding, an effect reversed by α₂ antagonists like yohimbine (Wellman, 2000). Nevertheless, findings regarding α-adrenergic receptor involvement in feeding are somewhat inconsistent, with reports of both feeding stimulation and null effects in different chicken strains. The β-adrenergic receptors have also been implicated, particularly in the suppression of meal consumption; central administration of β-agonists, including isoproterenol (a nonselective β₁/β₂ agonist) and selective β₂ and β₃ receptor agonists, reduces feeding in mammalian and avian models (Tachibana and Tsutsui, 2016, Baghbanzadeh et al., 2010, Kanzler et al., 2011). These variable but significant roles of adrenergic receptor subtypes in avian appetite regulation suggest that the adrenergic system acts as a crucial mediator in the anorexigenic action elicited by CCK-8s, warranting detailed investigation in neonatal broiler chicks.

The serotonergic system represents another essential neurochemical pathway involved in the central regulation of meal consumption. Serotonin (5-hydroxytryptamine), synthesized from the amino acid tryptophan primarily within neurons of the raphe nuclei, serves as a key neurotransmitter modulating diverse physiological functions, including appetite and energy homeostasis (Ligneul and Mainen, 2023). In avian species, dense serotonergic innervation has been identified in hypothalamic and diencephalic regions that orchestrate feeding behavior. Serotonergic receptor subtypes, particularly 5-HT2C and 5-HT1A, play critical roles in mediating anorexigenic and satiety signals (De Vry and Schreiber, 2000). Consistent with its established catabolic effects in mammals, central infusion of serotonin or serotonergic agonists reduces meal consumption in birds (Rahmani et al., 2022, Saadoun and Cabrera, 2002). Furthermore, emerging evidence underscores the complex interplay between the serotonergic and adrenergic systems in controlling avian feeding, suggesting receptor-level crosstalk in hypothalamic circuits (Zendehdel et al., 2017). Additionally, interactions between opioidergic and serotonergic pathways have been documented, with mu-opioid receptors implicated in mediating serotonin-induced suppression of feeding in neonatal broiler chicks (Rahmani et al., 2022). These findings underscore the complex and integral functions of the central serotonergic system in appetite regulation and support its investigation as a potential mediator of CCK-8s-induced hypophagia in our model.

Evidence from various models suggests potential mechanistic interactions between CCK, adrenergic, and serotonergic systems, such as CCK-facilitated serotonin release (Voigt et al., 1998), adrenergic enhancement of CCK secretion (Scott et al., 1996), and CCK modulation of adrenergic receptor binding (Kochman et al., 1984, Sullivan et al., 1994). However, whether such cross-talk occurs within central appetite circuits to mediate the anorexigenic effect of CCK-8s in broilers remains unknown. Therefore, this study was designed to systematically investigate the contributions of adrenergic (α1, α2, β1, β2) and serotonergic (5-HT1A, 5-HT2C) receptor subtypes to CCK-8s-induced hypophagia, by co-administering receptor-specific agents and measuring food intake.

## 2 Materials and methods

### 2.1 Birds

A cohort of 396 day-old Ross-308 broiler chicks was sourced from an accredited commercial hatchery (Mahan Company, Tehran, Iran). Upon delivery, the broilers were placed in thermostatically controlled brooders (HL-Heater, Qingdao Hrlynn Machinery Co, China) for acclimatization. Environmental conditions were maintained at 31 ± 1°C and 50 ± 5 % relative humidity, under a 23:1 h light/dark photoperiod. Light intensity was set between 25 and 30 lux. Following acclimatization, each chick was transferred to an individual wire-mesh cage (30 cm wide × 40 cm long × 40 cm high). Birds were offered a commercial starter diet sourced from Chineh Company, Tehran, Iran, with feed and water accessible ad libitum (Olanrewaju et al., 2007). To ensure precise measurement of feed intake, spilled feed was carefully collected from trays beneath cages and weighed; these amounts were subtracted from total offered feed to calculate accurate consumption. Freshly pre-weighed feed was provided immediately after ICV injections, and cumulative intake was measured at 30, 60, and 120 min post-infusion. Feed consumption data were standardized against individual body mass and reported as grams of intake per 100 g of body weight (g/100 g BW). Water was available throughout the experiment. All experimental protocols adhered strictly to the principles outlined in the Guide for the Care and Use of Laboratory Animals (NIH publication No. 85‑23, revised 1996) and complied with relevant national guidelines. The study received formal approval from the Institutional Animal Ethics Committee of the Faculty of Veterinary Medicine, University of Tehran (Approval No. IR.UT.VET.REC.1403.089).

### 2.2 Pharmacological agents

The pharmacological agents employed in this study comprised sulfated cholecystokinin octapeptide (CCK-8s; amino acids 26–33), alongside adrenergic and serotonergic modulators. Specifically, prazosin, an α1-adrenergic receptor antagonist; yohimbine, an α2-adrenergic receptor antagonist; metoprolol, a β1-adrenergic receptor antagonist; and ICI 118,551, a β2-adrenergic receptor antagonist, were utilized to delineate adrenergic receptor subtype contributions. Additionally, inhibitors and receptor agonist and antagonist targeting the serotonergic system included p-chlorophenylalanine (PCPA), a serotonin synthesis inhibitor; fluoxetine, a selective serotonin reuptake inhibitor (SSRI); 8-hydroxy-2-(di-n-propylamino)tetralin (8-OH-DPAT), a 5-HT1A receptor agonist; and SB-242,084, a selective 5-HT2C receptor antagonist. Evans blue dye was employed as a tracer for injection site verification (Sigma-Aldrich, USA).

All compounds were initially dissolved in absolute dimethyl sulfoxide (DMSO) before dilution in sterile 0.85 % saline containing Evans blue at a volumetric ratio of 1:250, resulting in a final DMSO concentration of 0.4 %. Previous investigations have demonstrated that this concentration of DMSO does not exert cytotoxic effects (Blevins et al., 2002). The vehicle control solution consisted of the DMSO/saline mixture including Evans blue to ensure consistent experimental conditions across treatment groups. All reagents and drugs were procured from Sigma-Aldrich (St. Louis, MO, USA) and Tocris Bioscience (Bristol, UK). The dosages of all pharmacological agents employed in this study were established based on prior empirical evidence and seminal investigations (Ebrahimnejad et al., 2025, Ashtari-Tavandashti et al., 2022). Table 1 presents the order and schedule of drug administration for the experimental groups.

**Table 1:** Sequence of pharmacological interventions in experimental groups.

**Experiments**	**Groups**			
	**A**	**B**	**C**	**D**
**1**	CS*	CCK-8s (0.25 nmol)	CCK-8s (0.5 nmol)	CCK-8s (1 nmol)
**2**	CS	Prazosin (10 nmol)	CCK-8s (1 nmol)	Prazosin + CCK-8s (10 nmol) + (1 nmol)
**3**	CS	Yohimbine (13 nmol)	CCK-8s (1 nmol)	Yohimbine + CCK-8s (13 nmol) + (1 nmol)
**4**	CS	Metoprolol (24 nmol)	CCK-8s (1 nmol)	Metoprolol + CCK-8s (24 nmol) + (1 nmol)
**5**	CS	ICI 118,551 (5 nmol)	CCK-8s (1 nmol)	ICI 118,551 + CCK-8s (5 nmol) + (1 nmol)
**6**	CS	PCPA (1.25 μg)	CCK-8s (1 nmol)	PCPA + CCK-8s (1.25 μg) + (1 nmol)
**7**	CS	Fluoxetine (10 μg)	CCK-8s (1 nmol)	fluoxetine + CCK-8s (10 μg) + (1 nmol)
**8**	CS	8-OH-DPAT (15.25 nmol)	CCK-8s (1 nmol)	8-OH-DPAT + CCK-8s (15.25 nmol) + (1 nmol)
**9**	CS	SB242084 (1.5 μg)	CCK-8s (1 nmol)	SB242084 + CCK-8s (1.5 μg) + (1 nmol)

CS: control solution (containing Evan’s blue); CCK-8s: sulfated cholecystokinin octapeptide; prazosin (α1 receptor antagonist); yohimbine (α2 receptor antagonist); metoprolol (β1 receptor antagonist); ICI 118,551 (β2 receptor antagonist); PCPA (parachlorophenylalanine, serotonin synthesis inhibitor); fluoxetine (serotonin reuptake inhibitor); 8-OH-DPAT (5-HT1A receptor agonist); SB242084 (5-HT2C receptor antagonist)**.**

### 2.3 Experimental design and intracerebroventricular injection procedure

The experimental design consisted of nine separate experiments, each including four groups with eleven birds per group (n = 44 birds per experiment). At the start of experiments, the average body weights (means ± SEM) of birds assigned to each group were as follows: Control, 45.2 ± 1.3 g; CCK-8s doses groups ranged from 44.8 ± 1.2 g to 45.5 ± 1.1 g; other pharmacological treatment groups showed similar balanced weights (all P ≥ 0.05 among groups). This ensured comparability in physiological status across treatments. A 3-hour food deprivation period was implemented prior to ICV injections while water remained accessible ad libitum. Central infusions were performed without anesthesia using a precision microsyringe (Hamilton, Switzerland), following the protocol described by Tachibana et al. (2022). This method is a well-established and widely accepted technique in neonatal poultry neuropharmacology (Saffar et al., 2025, Van Tienhoven and Juhasz, 1962). The brief restraint and rapid injection minimize distress, and the procedure is completed within seconds, causing no more discomfort than routine handling. The use of anesthesia itself can confound feeding studies by altering neurological and metabolic states, hence its omission is standard in this model. In brief, bird's head was firmly immobilized using a custom-designed acrylic apparatus, with the bill holder adjusted to a 45° angle, thereby positioning the calvarium in parallel alignment with the operating surface to facilitate precise intervention (Saffar et al., 2025, Van Tienhoven and Juhasz, 1962). A small access hole was drilled into a custom-designed plate aligned over the skull, directly above the right lateral ventricle, using stereotaxic coordinates relative to the cranial surface permitting precise needle insertion. The microsyringe needle was cautiously advanced 4 mm below the surface of the skull through the opening to deliver the injection. This technique is well documented to induce no physiological stress in young chicks (Saito et al., 2005). Each subject received a single central infusion of either vehicle or pharmacological agent at a fixed volume of 10 μL. Following infusion, broilers were immediately relocated to their respective cages and supplied with freshly pre-measured feed and water.

Although eleven birds per group were initially injected, only data from individuals exhibiting accurate dye placement within the lateral ventricle were included in the final analyses, resulting in 8–11 birds per group (see specific n-values in each figure caption). All experimental protocols were performed during the light period specifically from 08:00 AM to 3:30 PM, to control for potential diurnal variations in feeding behavior.

### 2.4 Statistical analysis

Data analysis was carried out utilizing a two-factor repeated measures ANOVA, considering Treatment and Time as within-subject factors in SPSS (v27.0). Following a significant interaction, Tukey’s multiple comparison test was employed for detailed post hoc analysis. Data are presented as mean ± SEM, with inferential significance accepted at the threshold of P < 0.05.

## 3 Results

### 3.1 Effects of CCK-8s on cumulative feed intake

**Fig. 1:**
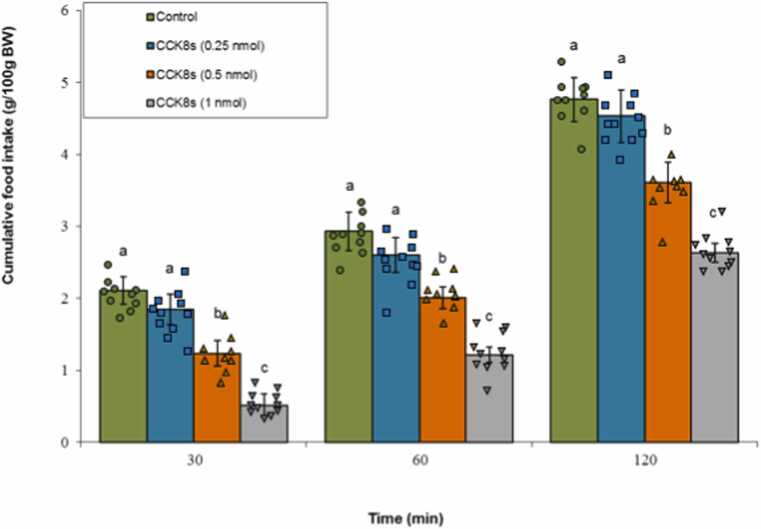
Effects of central infusion of vehicle or CCK-8s (0.25, 0.5, and 1 nmol) on cumulative food intake in broiler chicks. Values are mean ± SEM (Control, n = 10; 0.25 nmol, n = 11; 0.5 nmol, n = 9; 1 nmol, n = 11). Letters (a, b, c) mark statistically distinct groups (P < 0.05).

Central administration of CCK-8s at doses of 0.5 and 1.0 nmol significantly suppressed meal intake compared to the control treatment (P < 0.05), while the 0.25 nmol dose had no significant effect (P ≥ 0.05). ANOVA indicated statistically significant main effects for both the Treatment factor (F(3, 37) = 150.44, P < 0.001) and the Time factor (F(2, 74) = 1250.33, P < 0.001). Furthermore, a notable interaction effect was observed between Treatment × Time (F(6, 74) = 15.78, P < 0.001) (Fig. 1).

### 3.2 Role of adrenergic system

#### 3.2.1 α1-adrenergic receptor

**Fig. 2:**
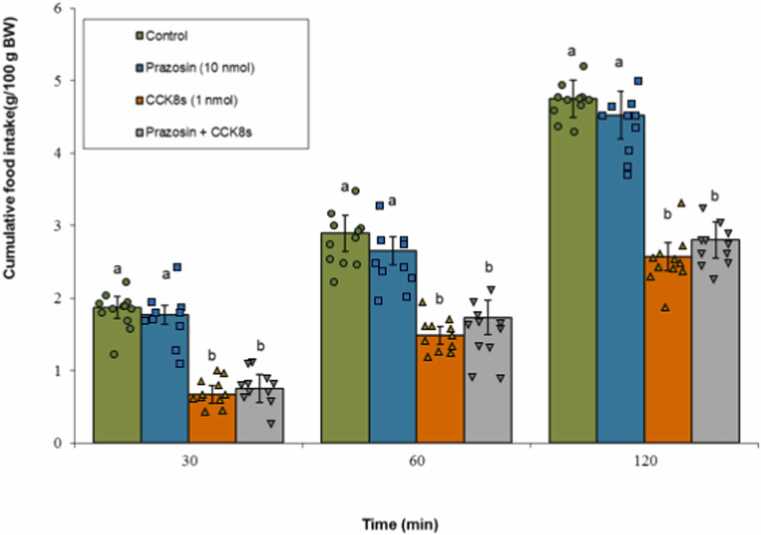
Effects of prazosin (10 nmol; α1-adrenergic antagonist), CCK-8s (1 nmol), or co‑administration on cumulative food intake. Values are mean ± SEM (Control, n = 11; prazosin, n = 10; CCK-8s, n = 11; combination, n = 11). Letters (a, b) mark statistically distinct groups (P < 0.05).

Administration of prazosin alone did not significantly influence feeding (P ≥ 0.05). CCK-8s (1 nmol) significantly reduced meal consumption (P < 0.05), and co-administration of prazosin with CCK-8s did not attenuate this anorexigenic effect (P ≥ 0.05). Statistical analysis identified significant primary effects for Treatment (F(3, 39) = 118.92, P < 0.001) and Time (F(2, 78) = 983.45, P < 0.001), as well as a statistically remarkable Treatment × Time interaction (F(6, 78) = 8.73, P < 0.001) (Fig. 2).

#### 3.2.2 α2-adrenergic receptor

**Fig. 3:**
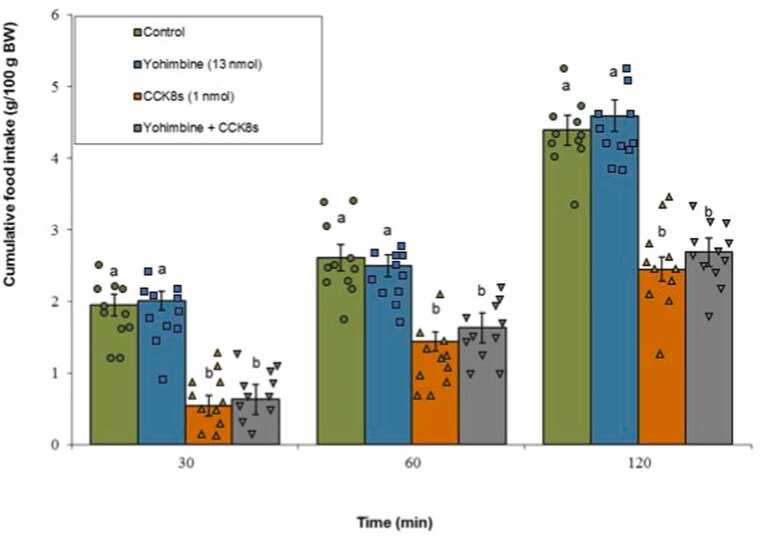
Effects of yohimbine (13 nmol; α2-adrenergic antagonist), CCK-8s (1 nmol), or co‑administration on cumulative food intake. Values are mean ± SEM (Control, n = 11; yohimbine, n = 11; CCK-8s, n = 11; combination, n = 11). Letters (a, b) mark statistically distinct groups (P < 0.05).

Yohimbine alone did not significantly affect meal consumption (P ≥ 0.05). CCK-8s significantly suppressed feeding behavior (P < 0.05), and the concomitant infusion of yohimbine with CCK-8s did not significantly alter the CCK-8s-induced anorexia (P ≥ 0.05). The ANOVA demonstrated statistically significant main effects for both Treatment (F(3, 40) = 165.33, P < 0.001) and Time (F(2, 80) = 1102.18, P < 0.001). Additionally, a significant interaction between Treatment ×Time was confirmed (F(6, 80) = 9.20, P < 0.001) (Fig. 3).

#### 3.2.3 β1-adrenergic receptor

**Fig. 4:**
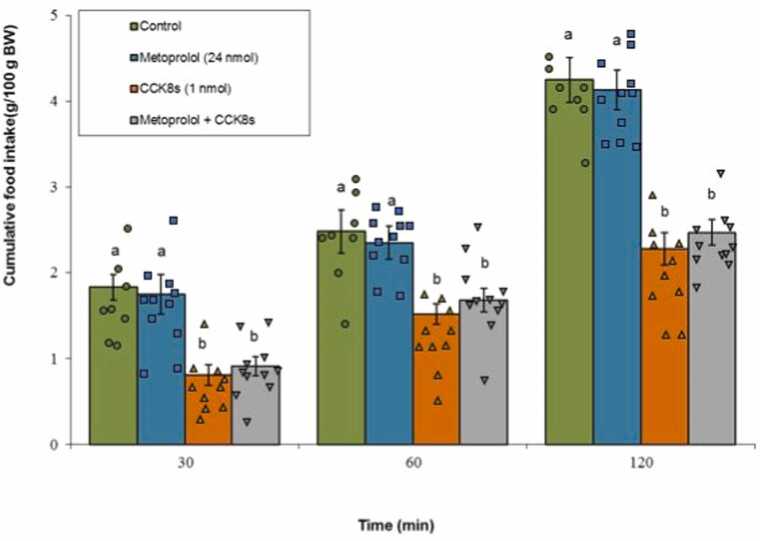
Effects of metoprolol (24 nmol; β1-adrenergic antagonist), CCK-8s (1 nmol), or co‑administration on cumulative food intake. Values are mean ± SEM (Control, n = 8; metoprolol, n = 11; CCK-8s, n = 10; combination, n = 11). Letters (a, b) mark statistically distinct groups (P < 0.05).

Metoprolol administered alone did not produce any remarkable changes in meal consumption (P ≥ 0.05). CCK-8s significantly suppressed feeding (P < 0.05), and the combined treatment of metoprolol and CCK-8s did not alter the CCK-8s-induced anorexia (P ≥ 0.05). The ANOVA results demonstrated a significant influence of both Treatment (F(3, 36) = 142.77, P < 0.001) and Time (F(2, 72) = 895.21, P < 0.001) as main factors. Furthermore, a statistically significant interactive effect between Treatment × Time was identified (F(6, 72) = 6.21, P < 0.001) (Fig. 4).

#### 3.2.4 β2-adrenergic receptor

**Fig. 5:**
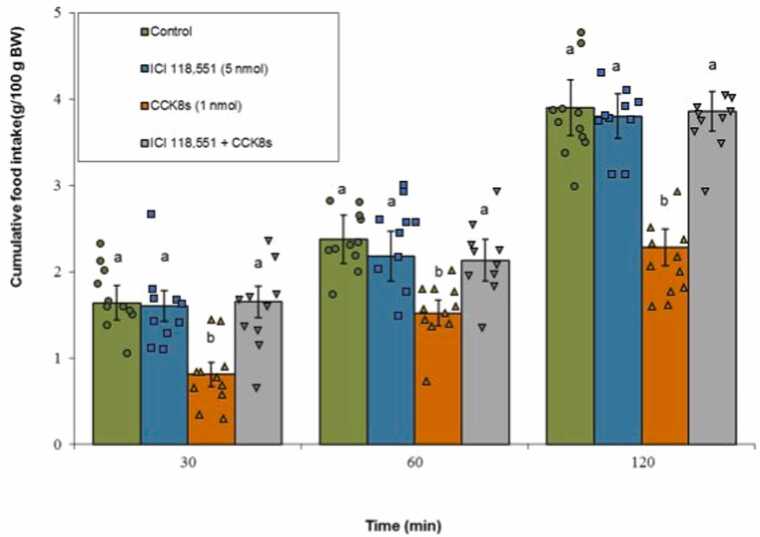
Effects of ICI 118,551 (5 nmol; β2-adrenergic antagonist), CCK-8s (1 nmol), or co‑administration on cumulative food intake. Values are mean ± SEM (Control, n = 11; ICI 118,551, n = 10; CCK-8s, n = 11; combination, n = 10). Letters (a, b) mark statistically distinct groups (P < 0.05).

The administration of ICI 118,551 alone did not significantly affect meal consumption (P ≥ 0.05). CCK-8s significantly suppressed feeding behavior (P < 0.05). Importantly, co-infusion of ICI 118,551 with CCK-8s notably attenuated the CCK-8s-induced anorexia (P < 0.05). Data analysis revealed a statistically notable impact of Treatment (F(3, 38) = 198.24, P < 0.001) and Time (F(2, 76) = 1050.89, P < 0.001). The interaction between Treatment × Time was also found to be significant (F(6, 76) = 8.95, P < 0.001) (Fig. 5).

### 3.3 Role of serotonergic system

#### 3.3.1 Serotonin synthesis inhibitor

**Fig. 6:**
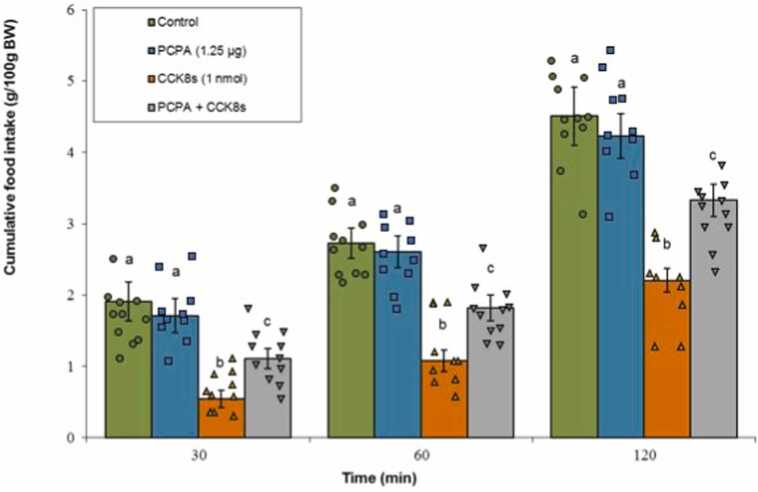
Effects of PCPA (1.25 μg; serotonin synthesis inhibitor), CCK-8s (1 nmol), or co‑administration on cumulative food intake. Values are mean ± SEM (Control, n = 11; PCPA, n = 10; CCK-8s, n = 9; combination, n = 11). Letters (a, b, c) mark statistically distinct groups (P < 0.05).

Administration of PCPA alone did not affect meal consumption (P ≥ 0.05). CCK-8s markedly suppressed feeding behavior (P < 0.05). Notably, the combined administration of PCPA with CCK-8s significantly attenuated the CCK-8s-induced anorexia (P < 0.05). ANOVA confirmed that both Treatment (F(3, 37) = 175.61, P < 0.001) and Time (F(2, 74) = 925.33, P < 0.001) exerted remarkable main effects. A notable Treatment × Time interaction was also detected (F(6, 74) = 6.12, P < 0.001) (Fig. 6).

#### 3.3.2 Serotonin reuptake inhibitor

**Fig. 7:**
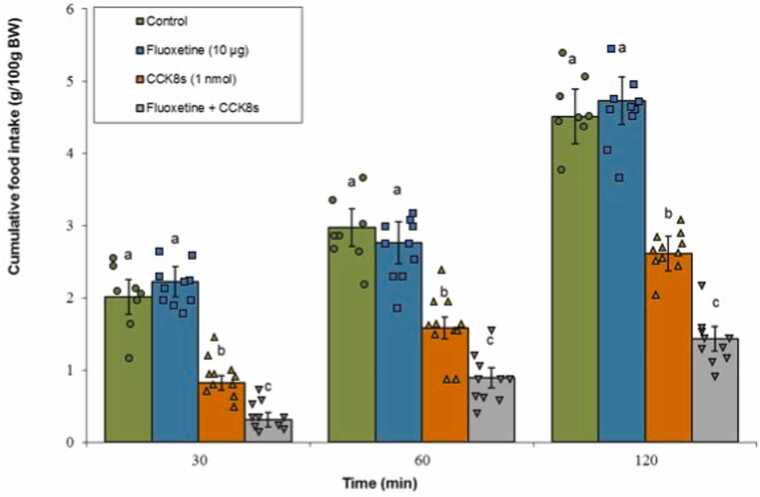
Effects of fluoxetine (10 μg; serotonin reuptake inhibitor), CCK-8s (1 nmol), or co‑administration on cumulative food intake. Values are mean ± SEM (Control, n = 8; fluoxetine, n = 10; CCK-8s, n = 11; combination, n = 10). Letters (a, b, c) mark statistically distinct groups (P < 0.05).

Fluoxetine administered alone had no significant impact on meal consumption (P ≥ 0.05). The infusion of CCK-8s significantly reduced feeding behavior (P < 0.05). When fluoxetine was co-administered with CCK-8s, the combined treatment produced an enhanced anorexigenic effect, with food intake notably lower than that of the CCK-8s alone group (P < 0.05). Statistical evaluation showed a significant influence of Treatment (F(3, 35) = 162.88, P < 0.001) and Time (F(2, 70) = 876.54, P < 0.001). Additionally, the interaction between these two factors was also statistically notable (F(6, 70) = 7.33, P < 0.001) (Fig. 7).

#### 3.3.3 5-HT1A receptor agonist

**Fig. 8:**
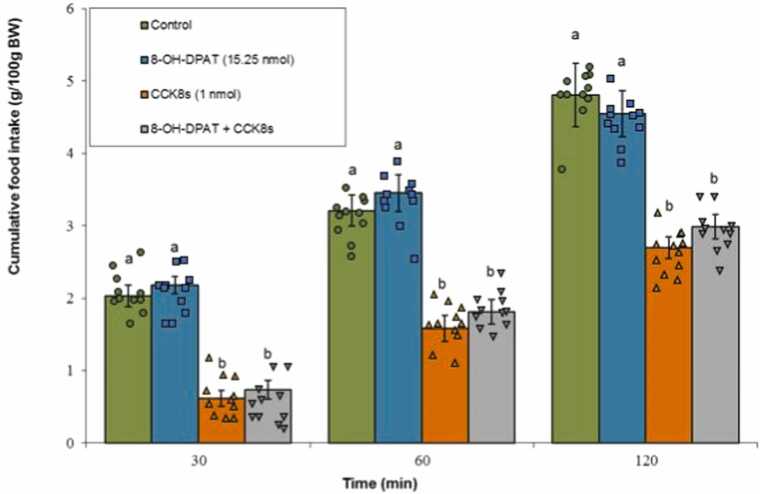
Effects of 8-OH-DPAT (15.25 nmol; 5-HT1A receptor agonist), CCK-8s (1 nmol), or co‑administration on cumulative food intake. Values are mean ± SEM (Control, n = 11; 8-OH-DPAT, n = 11; CCK-8s, n = 11; combination, n = 11). Letters (a, b) mark statistically distinct groups (P < 0.05).

Infusion of 8-OH-DPAT alone did not affect meal intake (P ≥ 0.05). Administration of CCK-8s significantly suppressed feeding (P < 0.05). The co-infusion of 8-OH-DPAT with CCK-8s did not modify the CCK-8s-induced anorexia significantly (P ≥ 0.05). The statistical analysis yielded remarkable main effects for both Treatment (F(3, 40) = 125.50, P < 0.001) and Time (F(2, 80) = 1005.92, P < 0.001), along with a notable Treatment × Time interaction (F(6, 80) = 7.90, P < 0.001) (Fig. 8).

#### 3.3.4 5-HT2C receptor antagonist

**Fig. 9:**
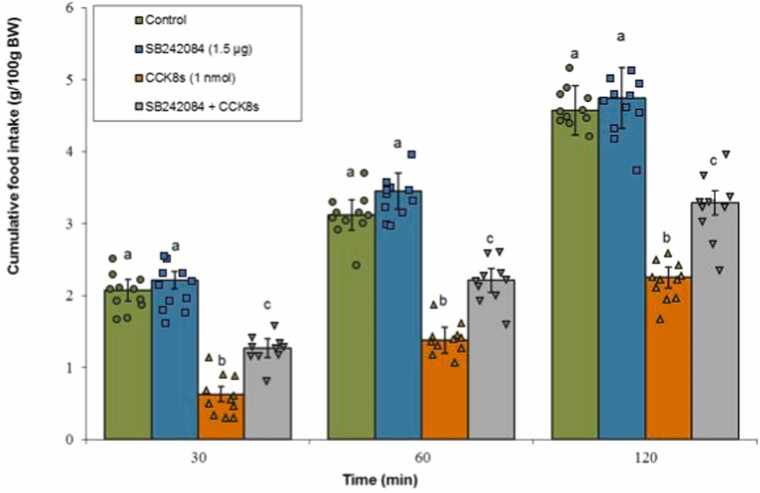
Effects of SB242084 (1.5 μg; 5-HT2C receptor antagonist), CCK-8s (1 nmol), or co‑administration on cumulative food intake. Values are mean ± SEM (Control, n = 11; SB242084, n = 11; CCK-8s, n = 11; combination, n = 10). Letters (a, b, c) mark statistically distinct groups (P < 0.05).

SB242084 administered alone did not produce remarkable changes in meal consumption (P ≥ 0.05). CCK-8s significantly suppressed feeding behavior (P < 0.05). Moreover, co-administration of SB242084 with CCK-8s attenuated the CCK-8s-induced anorexia significantly (P < 0.05). Statistical analysis indicated that Treatment (F(3, 39) = 182.45, P < 0.001) and Time (F(2, 78) = 955.67, P < 0.001) both had notable effects. A significant interactive effect between Treatment × Time was also evident (F(6, 78) = 9.01, P < 0.001) (Fig. 9).

## 4 Discussion

Understanding the neurochemical basis of appetite regulation in young broiler chicks is crucial for enhancing poultry production efficiency and animal welfare (Trocino et al., 2020). Central neuropeptides, including CCK, have emerged as key modulators of feeding behavior, yet the specific neural mechanisms and neurotransmitter systems involved remain incompletely defined in avian species. Our study advances this field by demonstrating that central infusion of CCK-8s induces a robust hypophagic response in neonatal broiler chicks. Importantly, we provide novel evidence that this anorexigenic effect is mediated through interactions with the central β2-adrenergic receptor and serotonergic signaling via 5-HT2C receptors, while other adrenergic and serotonergic receptor subtypes appear less involved. These findings deepen our mechanistic understanding of how CCK modulates feeding and reveal critical cross-talk between neurochemical systems regulating energy intake in developing birds.

### 4.1 Central hypophagic action of CCK-8s

Cholecystokinin, traditionally recognized for its peripheral digestive roles, also functions as an important central satiety peptide (Warrilow et al., 2023). Our results corroborate prior observations in avian models that central delivery of CCK-8s inhibited feed intake. Administration of 0.5 and 1 nmol notably decreased consumption for up to 120 min following injection, whereas the 0.25 nmol dose did not produce a measurable hypophagic response (Ebrahimnejad et al., 2025, Jelokhani et al., 2022). This threshold effect likely reflects receptor binding dynamics consistent with saturation of central CCKA (CCK1) receptors, which are the primary mediators of CCK's satiety effects (Staljanssens et al., 2011, Wan et al., 2023), distributed in hypothalamic and brainstem nuclei associated with feeding regulation (Tachibana et al., 2012, Wan et al., 2023). Unlike the relatively inert CCK-4 isoform, the biologically active peptide fragment CCK-8s elicits potent anorexia, consistent with reports in laying hens and other vertebrates (Ebrahimnejad et al., 2025, Gouda and Ganesh, 2025, Zahed et al., 2024).

This central hypophagic effect of CCK-8s appears rapid but partial, aligning with CCK's known role as an immediate 'meal termination' signal (Chandra and Liddle, 2025). Its ability to reduce both meal size and frequency suggests that CCK acts within discrete neural circuits that influence satiety perception and feeding drive. These circuits may integrate peripheral feedback from gastrointestinal mechanoreceptors and nutrient sensors (Lateef et al., 2011). Our data now extend this understanding by identifying specific neurotransmitter receptors that are critical for mediating this effect in broilers.

### 4.2 β2-Adrenergic receptors mediate CCK-8s hypophagia

Our investigations identify the β2-adrenergic receptor subtype as a critical mediator of CCK-8s-induced hypophagia. While selective antagonists for α1, α2, and β1 adrenergic receptors failed to modulate either food intake or the anorexic effect of CCK-8s, co-infusion of the β2 antagonist significantly attenuated CCK's suppression of feeding. This selective involvement of β2-receptors aligns with previous studies demonstrating β2-adrenergic contributions to appetite inhibition in avian and mammalian species (Alvarez-Crespo et al., 2025, Zendehdel et al., 2020). The β2-adrenergic receptor's expression in hypothalamic nuclei, including appetite-related regions such as the paraventricular nucleus and arcuate nucleus (ARC), supports its functional relevance (Moons et al., 1995, Oshima et al., 2014).

Mechanistically, β2-adrenergic receptor activation enhances adenylyl cyclase activity and cAMP production. This modulates downstream neuronal excitability and neurotransmitter release, which could potentiate satiety signaling cascades initiated by CCK (McGraw and Liggett, 2005, Shen et al., 2022). Notably, the inability of α-adrenergic receptor antagonists to suppress CCK's effects suggests a degree of specificity in adrenergic-cholecystokinin interactions, and indicates that these receptor subtypes may regulate feeding under alternative physiological states or through different neural pathways. This nuanced receptor subtype specificity adds complexity to adrenergic modulation of appetite, reflecting a balance of stimulatory and inhibitory influences.

### 4.3 5-HT2C-serotonergic receptors mediate CCK-8s hypophagia

Our findings also implicate the central serotonergic system, particularly 5-HT2C receptors, as pivotal in mediating CCK-8s' hypophagic effects. The suppression of CCK-induced anorexia by both PCPA and SB242084 strongly suggests that intact serotonergic signaling through 5-HT2C receptors is required. Conversely, the serotonin reuptake inhibitor fluoxetine enhanced CCK-8s hypophagia, indicative of a synergistic interaction where increased synaptic serotonin availability potentiates CCK's efficacy.

These results align with extensive mammalian literature describing 5-HT2C receptors as key regulators of satiety through their excitation of pro-opiomelanocortin (POMC) neurons located in the appetite regulation centers, suppressing orexigenic pathways (Asuku et al., 2024, Yao et al., 2021). In birds, although less studied, central serotonin similarly suppresses feeding, and 5-HT2C receptors have been identified as key modulators of meal consumption and reward pathways (Najafi et al., 2023, Zarei et al., 2024). The absence of significant effect by the 5-HT1A receptor agonist in our experiments further refines the receptor specificity of serotonergic involvement, emphasizing the unique functional contribution of 5-HT2C subtype in chick satiety signaling.

### 4.4 Integrated neurochemical cross-talk among CCK and adrenergic and serotonergic systems

CCK, β2-adrenergic, and serotonergic receptors are co-localized in key hypothalamic and brainstem regions, such as the nucleus of the solitary tract and ARC (Moons et al., 1995, Kang et al., 2009, Voits et al., 1996). This anatomical overlap supports a biologically plausible model of neurochemical integration. In this model, CCK modulates feeding through a network that involves 5-HT2C and β2-adrenergic receptors. Previous research demonstrate that CCK facilitates serotonin release and activates serotonergic neurons (Voigt et al., 1998). Additionally, β-adrenergic receptors have been identified in the secretory intestinal tumor cell line (STC-1), where they may enhance CCK release via a calcium-dependent mechanism (Scott et al., 1996). Conversely, CCK does not seem to engage directly with adrenergic receptors; rather, it may influence β-adrenergic receptor binding affinity via indirect mechanisms (Kochman et al., 1984).

Clinical and animal studies reinforce this cross-talk, showing that disruption of either serotonergic or CCK receptor signaling alters meal consumption and energy balance (Cooper et al., 1990, Grignaschi et al., 1993). CCK's inhibition of α2-adrenergic receptor-induced analgesia and its activation of preproglucagon neurons through adrenergic and glutamatergic inputs illustrate the complexity of these intersecting pathways in central homeostatic regulation (Sullivan et al., 1994, Hisadome et al., 2011).

Our data extend these findings to neonatal broiler chicks, providing direct pharmacological evidence that CCK-8s interacts synergistically with β2-adrenergic and serotonergic (5-HT2C) circuits to fine-tune satiety responses.While our results emphasize adrenergic and serotonergic pathways, it is possible that other neurotransmitter systems, such as dopaminergic or opioidergic circuits, also contribute to CCK-8s-induced anorexia (Rahmani et al., 2022, Ashtari-Tavandashti et al., 2022).

### 4.5 Limitations

While our study elucidates acute central mechanisms, we acknowledge limitations including the absence of direct molecular measurements of receptor and neuropeptide regulation, restricted observation to short-term feeding effects, and the use of a single broiler. Consequently, the generalizability of these findings to practical field conditions, where environmental variables, diet composition, and long-term growth dynamics differ, requires further investigation. Moreover, neuroanatomical localization of receptor co-expression and neuronal activation was beyond the scope of this study but remains a critical next step.

### 4.6 Implications and future directions

The identification of β2-adrenergic and 5-HT2C receptor involvement in CCK-8s-induced hypophagia informs potential strategies to manipulate feeding in commercial broiler chicks, aiming to improve feed efficiency and growth performance through neuroendocrine modulation. Targeted activation or sensitization of these pathways could refine satiety signals, reduce overeating, and minimize feed wastage, aligning with sustainability goals.

A key direction for upcoming research involves assessing the sustained metabolic consequences of altering CCK and neurotransmitter receptor signaling, and employ molecular and neuroanatomical methods (e.g., receptor expression profiling, c-Fos immunohistochemistry) to map activated neural circuits. Studies exploring peripheral-central feedback mechanisms, including vagal afferent signaling and gut hormone interactions, will further elucidate integrated appetite control networks in poultry.

## 5 Conclusion

In sum, our findings demonstrate that central infusion of CCK-8s evokes potent hypophagia in neonatal broiler chicks through mechanisms involving β2-adrenergic and serotonergic (5-HT2C receptor) systems. This neurochemical interplay highlights species-specific adaptations in avian appetite regulation and offers a compelling framework for future investigations and translational applications in feed management. By advancing the neuroendocrine understanding of feeding control in poultry, these results contribute to optimizing animal production and welfare in a sustainable manner.

## CRediT authorship contribution statement

**Emad Shahrabi:** Writing – review & editing. **Keyvan Hasani:** Writing – review & editing. **Ghazaleh Mahdavi:** Writing – review & editing, Investigation, Formal analysis. **Hamed Zarei:** Writing – review & editing, Writing – original draft, Conceptualization.

## Ethical approval

All procedures were conducted in compliance with the Guide for the Care and Use of Laboratory Animals (NIH, publication No. 85–23, revised 1996) and approved by the Institutional Animal Ethics Committee of the Faculty of Veterinary Medicine, University of Tehran (Approval No. IR.UT.VET.REC.1403.089).

## Declaration of Generative AI and AI-assisted technologies in the writing process

During the preparation of this work the author(s) used Perplexity AI in order to identify and correct potential grammatical errors and improve the overall flow and readability of the manuscript. After using this tool, the author(s) reviewed and edited the content as needed and take(s) full responsibility for the content of the published article.

## Funding

This research did not receive any specific grant from funding agencies in the public, commercial, or not-for-profit sectors.

## Conflicts of Interest

The authors declare that they have no known competing financial interests or personal relationships that could have appeared to influence the work reported in this paper.

## Data Availability

All data generated or analyzed during this study are included in this published article.
